# Taurolidine-citrate lock solution (TauroLock) significantly reduces CVAD-associated grampositive infections in pediatric cancer patients

**DOI:** 10.1186/1471-2334-8-102

**Published:** 2008-07-29

**Authors:** Arne Simon, Roland A Ammann, Gertrud Wiszniewsky, Udo Bode, Gudrun Fleischhack, Mette M Besuden

**Affiliations:** 1 Pediatric Hematology and Oncology, Children's Hospital Medical Center, University of Bonn, Germany; 2 Pediatric Hematology and Oncology, Department of Pediatrics, University of Bern, Switzerland

## Abstract

**Background:**

Taurolidin/Citrate (TauroLock™), a lock solution with broad spectrum antimicrobial activity, may prevent bloodstream infection (BSI) due to coagulase-negative staphylococci (CoNS or 'MRSE' in case of methicillin-resistant isolates) in pediatric cancer patients with a long term central venous access device (CVAD, Port- or/Broviac-/Hickman-catheter type).

**Methods:**

In a single center prospective 48-months cohort study we compared all patients receiving anticancer chemotherapy from April 2003 to March 2005 (group 1, heparin lock with 200 IU/ml sterile normal saline 0.9%; Canusal^® ^Wockhardt UK Ltd, Wrexham, Wales) and all patients from April 2005 to March 2007 (group 2; taurolidine 1.35%/Sodium Citrate 4%; TauroLock™, Tauropharm, Waldbüttelbrunn, Germany).

**Results:**

In group 1 (heparin), 90 patients had 98 CVAD in use during the surveillance period. 14 of 30 (47%) BSI were 'primary Gram positive BSI due to CoNS (n = 4) or MRSE (n = 10)' [incidence density (ID); 2.30 per 1000 inpatient CVAD-utilization days].

In group 2 (TauroLock™), 89 patients had 95 CVAD in use during the surveillance period. 3 of 25 (12%) BSI were caused by CoNS. (ID, 0.45). The difference in the ID between the two groups was statistically significant (P = 0.004).

**Conclusion:**

The use of Taurolidin/Citrate (TauroLock™) significantly reduced the number and incidence density of primary catheter-associated BSI due to CoNS and MRSE in pediatric cancer patients.

## Background

The use of long-term central-venous access devices (CVAD, Port- or Broviac-/Hickman catheter type) contributed to the success of intensive chemotherapy in pediatric oncology, but it also increased the risk for infection [1-3]. Catheter-related infections result in an increased length of hospital stay and in higher costs [4,5]. They drain resources for nursing care, antimicrobials and for surgical removal of the device in those cases, which do not respond to antimicrobial treatment. Since most of these infections are treated with glycopeptides, they contribute to the overall selective pressure on glycopeptide-resistant enterococci [6]. Catheter associated infections may occur in patients irrespective of neutropenia; in a multicenter study from Italy 68 of 191 (36%) patients with catheter associated bloodstream infection did not display neutropenia at the time of the diagnosis [3]. In principle, the catheter-related bloodstream infections (BSI) may result from contamination/colonization of the catheter hub or of the internal surface of the catheter lumen, i.e. from the hands of health-care workers or contaminated infusion fluids. It may further be related to external surface (skin) colonization originating at the CVAD exit site [7]. In addition, a CVAD-related infection may be acquired via the blood stream, e.g., due to bacterial translocation in patients with chemotherapy induced mucositis [8] or from a distant focus of infection [9].

The majority of the hub- and the intraluminal infections without any other apparent source are caused by coagulase-negative staphylococci (CoNS) [2]. The recently finalized German surveillance study for nosocomial infections in pediatric cancer patients [10] identified CoNS as causative pathogen in 50 of 138 (36%) bloodstream infections; 38% of the CoNS isolates were resistant to methicillin (MRSE). In the Italian survey of Viscoli et al., 43 of 191 (23%) of all BSI were caused by CoNS [3].

Taurolidine is a chemically modified amino acid (taurin) with broad spectrum antimicrobial activity in vitro [11-14]. Toores-Vierra at al. [14] confirmed the in vitro activity of taurolidine against a broad range of *Gram *positive and *Gram *negative organisms including oxacillin-resistant *S. aureus *and CoNS, vancomycin-resistant enterococci, and *Gram *negative pathogens, including *P. aeruginosa *and *Stenotrophomonas maltophilia*; the MIC 90 results of most isolates were at least 10 times lower than the concentration of the Taurolidine-preparation used in this study (13,5 mg/ml).

A small series of studies investigated the use of taurolidine in the prevention and (adjuvant) treatment of CVAD-related infections in dialysis [15,16], long-term parenteral nutrition [17,18] and as adjunctive treatment in CVAD infections in adult cancer patients [19]. Two potential advantages attributed to its use are

(1) taurolidine may prevent the formation of biofilm on the internal surface of the catheter and inside the subcutaneous reservoir of infusion ports [20,21].

(2) no resistance against taurolidine has to be expected in clinically relevant isolates [12-14,22,23].

Furthermore, taurolidine does not display any significant toxicity even after high dose intravenous infusion [24].

The prospective 48-months cohort study presented here investigated the impact of a taurolidine/citrate containing CVAD lock solution on catheter-associated infections in a pediatric oncology unit at the Children's Hospital Medical Center, University of Bonn, Germany.

## Methods

### Setting

The pediatric oncology unit of the University Children's Hospital in Bonn, Germany, is a 17-bed tertiary care facility providing inpatient care for 900 admissions during about 5,000 inpatient days (~50 newly diagnosed pediatric cancer patients) per year. The CVAD clinical-practice recommendations of the German Society of Pediatric Oncology and Hematology (GPOH) have been coordinated by one of the authors (AS), are strictly followed during in- and outpatient care, and fully match the CDC-Recommendations [25] with the following exceptions: octendine 0.1%/phenoxyethanol 2% (Octenisept^®^, Schuelke & Mayr, Norderstedt) is used for local antisepsis [26,27]. In addition, intravenous administration sets are changed routinely only once a week [28] unless they have been used for lipid infusion (once a day) or for blood product administration (6 hours after the transfusion) [29]. In this pediatric oncology unit, totally implanted port catheters are preferably used in patients with conventional chemotherapy regimens (median duration of neutropenia <7 days). Double-lumen Broviac/Hickman catheters are used in patients for whom an intensive and complicated treatment course or a stem cell transplantation is anticipated. As a result of this policy, patients have ports in place in 60–70% and Broviacs in 30–40% of all inpatient treatment days.

### Patients, inclusion and exclusion criteria

All patients with cancer and a long-term intravenous access device (CVAD) were eligible. Patients with short term non-tunnelled central venous catheters and patients with hematological diseases without neutropenia (i.e. Blackfan-Diamond or sickle cell anemia) were excluded. CVAD-related bloodstream infections (BSI) and all episodes of fever were prospectively evaluated by a study nurse and the principal investigator (pediatric oncologist and infectious disease consultant).

The following two groups in two consecutive 48-month surveillance periods were compared: all patients receiving anticancer chemotherapy from April 2003 to March 2005 (group 1, 200 IE heparin lock in 2 ml sterile normal saline 0.9%; Canusal^® ^Wockhardt UK Ltd, Wrexham, Wales, U.K.) and all patients from April 2005 to March 2007 (group 2; taurolidine 1.35%/Sodium Citrate 4%; TauroLock™, Tauropharm, Waldbüttelbrunn, Germany). Six patients first studied in group 1 were excluded from analysis in group 2 despite the later use of TauroLock™. Both lines of the double-lumen Broviac catheters were flushed and relocked in our outpatient clinic or at home by members of our outpatient pediatric oncology care team one to two times a week. Ports were never flushed while not in use (no puncture of the port just to flush and re-lock the lumen). TauroLock™ was also used for intermittent locks in patients, who were disconnected from the infusion system during inpatient stay, but this was only rarely the case. Patients with clinical signs of infection around the exit site of the CVAD (at the site of needle access in Ports) were counted as secondary BSI, if the same bacterial species with identical in vitro sensitivity results was detected in blood cultures and in local wound swabs. In case of a remove or change of the CVAD, the reason for this intervention was documented. Since in some patients the anticancer treatment period crossed the time frame of the two surveillance periods and as some patients had to receive more than one subsequent CVAD, patients were allowed to be included into both surveillance periods and without restriction on the number of CVADs subsequently used; every CVAD was counted separately. Neutropenia was defined as an absolute neutrophil count < 0.5 × 10^9^/l or a leukocyte count < 1 × 10^9^/l in absence of a differential WBC. Except cotrimoxazole for the prevention of *Pneumocystis jirovecii *pneumonia, no antibiotic prophylaxis/selective decontamination regimen was administered to the patients with hematologic malignancies or autologous stem cell transplantation.

### Microbiological methods and interpretation of results

Two blood cultures were collected in all patients with fever (temperature >38,5°C for at least 4 hours or once >39°C) under aseptic conditions before the first dose of antibiotics and were routinely tested according to standard procedures [30]. In the taurolidine group, the (first) 2 ml proportion of the aspirate, which included the lock, was discarded; otherwise, false negative cultures might have been the result of the antimicrobial effect of taurolidine added to the Bactec^® ^culture system (Becton Dickinson GmbH, Heidelberg, Germany).

According to the recommendations of the German Society of Pediatric Hematology and Oncology [31,32], no concomitant peripheral blood cultures were investigated.

A 'primary CVAD-related *Gram *positive BSI' was allocated to any patient who had a CVAD in place, clinical signs of infection plus at least two positive blood culture bottles for CoNS or MRSE taken from a CVAD, and no evidence of another primary focus of infection.

In our pediatric oncology unit, a prospective surveillance system, based on CDC's National Nosocomial Infections Surveillance (NNIS) methods, has been continuously used since 1998. Methods and results of this surveillance protocol have been published previously [10,33].

### Statistical analysis

Since continuously measured data were non-normally distributed, median and interquartile range (IQR) was calculated, and exact nonparametric analytical methods (Fisher's test, Fisher-Freeman-Halton test, Wilcoxon-Mann-Whitney test) were applied. Incidence densities and their exact 95% confidence intervals were calculated as Poisson event rates, and compared by testing for homogeneity of rates. All analyses were calculated as two-sided tests, and P-values < 0.05 were considered to be statistically significant.

### Ethic approval and informed consent

The study protocol was approved by the ethics committee of the medical faculty, University of Bonn and by the German Society of Infectious Diseases in Childhood (DGPI). Informed consent to participate in the surveillance study was given by all patients or their legal guardians.

## Results

In total, 179 patients were studied. The two resulting study groups of 90 and 89 patients, respectively, were comparable as to basic characteristics like age, underlying illness and relapse status (Tab. 1).

**Table 1:** Basic patient characteristics and blood stream infections (BSI).

Item	Group 1 (heparin)	Group 2 (TauroLock™)	P value
No. of patients	90	89	-
Male (proportion in %)	51 (57)	60 (67)	0.16
Female (proportion in %)	39 (43)	29 (33)	
Age (years): median	10.4	7.2	0.52
IQR^Δ^	5.2 to 14.7	3.7 to 16.1	
Range	0.2 to 35.2	0.0 to 35.4	
CVAD utilization days			
Cumulative No.	6,086	6,705	#
Port	3,672	3,989	
Broviac	2,414	2,716	
Malignancy No. (%)			0.09
ALL	21 (23)	26 (29)	
AML	3 (3)	6 (7)	
NHL and HD	15 (17)	7 (8)	
Solid tumor	30 (33)	22 (25)	
ZNS	18 (20)	27 (30)	
MAS	2 (2)	0 (0)	
MDS	0 (0)	1 (1)	
LCH	1 (1)	0 (0)	
Malignancy in relapse No. (%)	16 (18)	13 (15)	0.69
No of patients with			0.41
Port (proportion in %)	68 (76)	62 (70)	
Broviac (proportion in %)	22 (24)	27 (30)	
CVAD removal due to infection: No. (%)	4 (4.4)	3 (3.4)^Ω^	1.00

Group 1 = heparin (2003–2005) vs. group 2 = TauroLock™ (2005–2007)

MAS Hemophagocytic lymphohistiocytosis, MDS myelodysplastic syndrome,

LCH Langerhans Cell Histiocytosis.

* CoNS = Coagulase-negative staphylococci.

Δ IQR = Interquartile range, 25–75. Percentile

# Utilization days per individual patient were not available; thus, P-values could not be calculated.

Ω None of these due to *Gram *positive infections caused by CoNS or MRSE

### Group 1 (2003–2005) Heparin

In group 1 (heparin), 90 patients had 98 CVAD in use during the surveillance period. In 8 patients (9%) the CVAD had to be changed. Of the initially implanted 90 CVAD, 4 (4.4%) had to be removed because of an infection; of these, 3 because of a primary *Gram *positive BSI (MRSE) in Port catheters (3.3%). In addition, 4 patients experienced mechanical complications (accidental removal, dislocation or occlusion).

**Figure 1:**
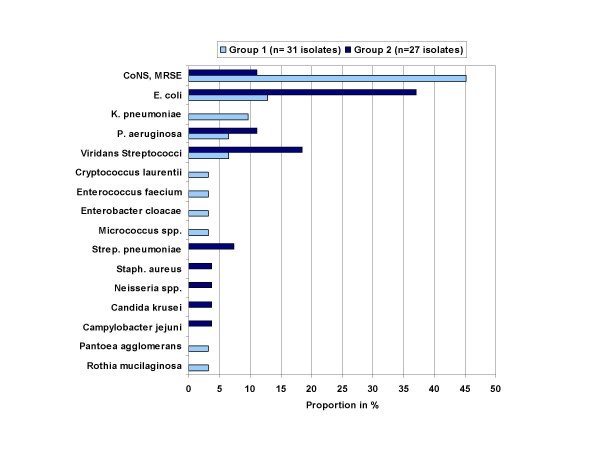
**Distribution (in %) of blood culture isolates**. [Group 1 heparin; 2003–2005 30 BSI; group 2 TauroLock™; 2005–2007; 25 BSI).

Thirty blood culture-positive BSI were documented in 24 (27%) of the 90 patients in group 1 (Tab. 2 and Fig. 1). Of these, 14 (47%) were allocated to the category 'primary *Gram *positive BSI due to CoNS (n = 4) or MRSE (n = 10)'. The cumulative duration of inpatient CVAD utilization in group 1 was 6086 days (3672 days for Ports; 2414 days for Broviacs). Thus, the incidence density (number of events per 1000 inpatient CVAD utilization days) for primary *Gram positive *BSI due to CoNS or MRSE was 2.30. No patient died related to the infection.

**Table 2:** Evaluation of blood stream infections.

Item	Group 1 (heparin)	Group 2 (TauroLock™)	P value
No. of BSI events	30	25	
No. (%) of patients with at least 1 BSI	24 (27)	21 (24)	0.74
No. (%) of BSI with CoNS* or MRSE^§^	**14 (47)**	**3 (11)**	**0.004**
	CoNS: 4 (13)	CoNS: 3 (11)	
	MRSE: 10 (33)	MRSE: 0 (0)	
Incidence density^‡ ^for All BSI events (CI_95_)	4.93 (3.33–7.04)	3.82 (2.52–5.56)	0.35
Incidence density^‡ ^calculated with the number of specific isolates			
**BSI with CoNS/MRSE**	**2.30 (1.26–3.86)**	**0.45 (0.09–1.31)**	**0.004**
BSI other Gram positive	0.66 (0.18–1.68)	1.19 (0.52–2.35)	0.32
BSI *E. coli*	0.66 (0.18–1.68)	1.49 (0.72–2.74)	0.15
BSI all Gram negative	1,97 (1.02–3.44)	2.24 (1.25–3.69)	0.74

Group 1 = heparin (2004–2005) vs. group 2 = TauroLock™ (2005–2006)

* CoNS = coagulase-negative Staphylococci (methicillin-sensitive)

§MRSE = methicillin-resistant coagulase-negative Staphylococci

+ The percentage refers to all documented BSI in this group (100%).

‡ The ID refers to the number of events per 1000 inpatient CVAD utilization days.

Δ IQR = interquartile range, 25–75. Percentile

### Group 2 (2005–2007) Taurolidine

In group 2 (TauroLock™), 89 patients had 95 CVADs in place during the 24-months surveillance period. In 6 patients (7%) more than one CVAD had to be implanted subsequently. No CVAD had to be removed because of a 'primary *Gram *positive BSI due to CoNS or MRSE'. Three (3%) had to be removed because of an infection: one port with local wound infection (*S. aureus*);, one Broviac with local wound infection (*Pseudomonas aeruginosa*), one Broviac after detection of *Candida krusei *in blood cultures. In 3 patients the CVAD was removed following mechanical complications.

Twenty-five blood culture-positive bacterial BSI were documented in group 2 (Tab. 2 and Fig. 1). Of these, 3 (12%) were due to CoNS, allocated to the category 'primary *Gram *positive BSI with CoNS or MRSE'. In one of these cases the Port-needle had been set under the circumstances of an acute life threatening emergency (shock) without sufficient skin antisepsis. In another patient, a 16 year old female with AML and mucositis, the mother had re-locked the Broviac at home. On the day of admission with fever and neutropenia, antibiotic treatment had to be switched from piperacillin-tazobactam to meropenem, amikacin, teicoplanin due to clinical sepsis. The attending physicians did not allocate these symptoms to the multi-susceptible CoNS detected in the initial blood cultures.

The cumulative duration of CVAD utilization in group 2 was 6705 days (3989 days for Port; 2716 days for Broviac). Thus, the incidence density (per 1000 CVAD utilization days) for primary *Gram positive *BSI due to CoNS or MRSE was 0.45 (95% CI, 0.09 to 1.31). This was significantly lower than the respective incidence density in group 1 (p = 0.004; Table 2).

No patient died related to the infection.

There was a non-significant trend towards a higher incidence density of *E. coli *BSI in group 2 versus group 1 (Table 2; 10 vs. 4 infections; incidence density 1.49 vs. 0.66, P = 0.15). This prompted us to take a closer look at these cases in group 2. The events were not temporally related (no outbreak).

Five patients with *E. coli *BSI in group 2 received intensive (re-) induction treatment for acute leukemia. One 17 year-old girl with AML in relapse experienced 3 subsequent *E. coli *BSI without any clinically documented source. One patient, a 5 year-old boy with ALL and Down's syndrome developed a deep local wound infection after bone marrow puncture in the diaper area, which probably represented the source of secondary bacteremia. In one case, the bacteremia was related to a urinary tract infection (ALL, female, 16 months).

### Safety and convenience issues

No hypersensitivity reactions and no clinical or laboratory signs of hypocalcaemia were observed. Some patients complained about the sensation of an uncommon taste directly after the injection of TauroLock™ when the CVAD was just flushed without previous aspiration of the lock. None of the adolescent patients, who were able to communicate about this minor adverse effect, refused to continue the TauroLock™ prophylaxis. In one patient, TauroLock™ was inadvertently used to lock a peripheral venous access device. The patient immediately complained about severe local pain. The venous access device was flushed with normal saline and remained in place without local signs of phlebitis or extravasation.

## Discussion

In our pediatric oncology unit, the routine use of taurolidine 1.35%/sodium-citrate 4% as a lock solution in patients with long-term CVAD resulted in a statistically significant and clinically relevant decrease in the incidence density of primary *Gram *positive CVAD-associated infections due to CoNS and MRSE. In accordance with single centre studies performed in patients with hemodialysis catheters [15,16], our results demonstrate the efficacy of the taurolidine lock solution. While the sequential methodology of our study may not fully delineate the impact of this intervention; a prospective randomized double-blind study would be expedient for external validation.

If one takes the many different possible origins of BSI in immunocompromised pediatric cancer patients into consideration, it seems difficult to calculate, which proportion of all infections may be preventable through an intervention, which aims only at the intraluminal colonization of the device. Gaur et al. (St. Jude Children's Research Hospital, Memphis), recently investigated infectious complications in pediatric cancer patients in a series of studies with sophisticated microbiological methods to confirm the catheter as the primary site of infection. They came to the conclusion that 36% (21 of 59) of all blood culture-positive infections were definitely related to the CVAD-lumen [34,35]. We did not cultivate the first two ml of the blood sample drawn from the CVAD, which contained the taurolidine lock at 13.5 mg/ml (2 ml) in order to avoid false negative results due to the antimicrobial effect of taurolidine in the blood culture bottle. Due to the high in vitro activity of taurolidine at 10 times lower concentrations it is highly improbable, that cultivation of these 2 ml would have changed the results.

Our prospective surveillance study of CVAD-associated infections revealed that the relative risk of a CVAD-associated infection is up to 21 times higher in inpatients (p < 0.01) [33]. This is the unfavorable consequence of multiple manipulations and prolonged 'hands-on time' during inpatient care with blood drawings, changes of the administration sets [28], administration of chemotherapy, antibiotics, pain medication, parenteral nutrition [36], and blood products [37]. Taurolidine can only display its antimicrobial activity in a CVAD which is actually locked. Therefore, many opportunities during injection and infusion or blood drawing activities remain in clinical practice to contaminate the device and subsequently infect the patient. It remains unknown whether an 'intermittent-lock approach' with taurolidine (e.g. for 4 hours) would result in a significant benefit in inpatients.

Although not statistically significant, there have been more blood stream infections with *E. coli *observed in the second surveillance period. A thorough investigation of each case of *E. coli *BSI did not reveal any plausible relation to the use of TauroLock ™. Taurolidine is active against *E. coli *in vitro [13,14]. Most of the patients with *E. coli *BSI (as those with BSI due to viridans streptococci) were intensively treated for acute leukemia, and faced an increased risk of gastrointestinal translocation due to mucositis and prolonged neutropenia. In the last 5 years, *E. coli *has been the most prevalent *Gram *negative bacterial species detected in blood cultures. This may be the case, because we do not use selective decontamination regimens [38] nor any antibacterial prophylaxis against *Gram *negative bacteria. Due to the historical control group design of our study, we can only speculate, that in group 2 at about 10 prevented *Gram *positive infections would have been observed in addition to the *Gram *negative ones without the prophylactic use of the taurolidine lock solution. This issue should be addressed in a prospective randomized study.

In our unit, the mean charges for the management of only two BSI events (~€ 5000 per BSI) compensate the higher 12-month acquisition cost (TauroLock™ vs. Canusal™) for the whole patient population. From an economical perspective, the prevention of a single episode would be sufficient, if Broviac CVAD were flushed and relocked always only once a week [32].

Potential complications (hypersensitivity and hypocalcaemia) seem to be very unlikely, since the minimal injected amount of taurolidine is readily metabolized to taurin and the ≤ 2.5 ml Citrate 4% injection is rapidly diluted in the vena cava superior, in particular in case of a slow administration. TauroLock™ must not be used to lock peripheral venous catheters.

In contrast to a previous study in dialysis patients [15] which described a lower rate of unassisted catheter patency (without tissue plasminogen activator instillation) among patients, who received taurolidine, than among control patients (32% vs. 76%; P < .001), we could not detect significant differences between the two groups considering catheter occlusions (2 in each group) or catheter related thrombotic events (one in group 1).

## Conclusion

This 48 months prospective cohort study from a pediatric oncology unit showed that the use of Taurolidine 1.35%/Sodium-Citrate 4% (TauroLock™) as standard lock solution in long-term CVADs significantly reduced the incidence density of CVAD-associated infections due to CoNS or MRSE. The described reduction of infectious events reveals an insistent argument to perform a prospectively randomized, double-blinded, multicenter study including a sufficient number of pediatric patients with long term CVAD.

## Competing interests

The authors declare that they have no competing interests.

## Authors' contributions

AS and RAA performed the data analysis and wrote the manuscript; GW was responsible for the primary data management (study nurse); UB and GF contributed substantially to the final version of the manuscript. AS and MMB designed the study protocol and were the responsible attending physicians. All authors read and approved the final manuscript.

## Pre-publication history

The pre-publication history for this paper can be accessed here:


